# Supplementary material for: Novel, Blended Polymeric Microspheres for the Controlled Release of Methotrexate: Characterization and In Vivo Antifibrotic Studies
Source: Bioengineering (Basel). 2023 Feb 27;10(3):298. doi: 10.3390/bioengineering10030298 (PMC10045572; doi:10.3390/bioengineering10030298)
Supplement: Supplementary file 1 [file bioengineering-10-00298-s001.zip › bioengineering-2205156-supplementary.pdf]

1.1 Fibroblast culture

Foreskin samples, obtained from infants being circumcised, were collected individually in Dulbecco's modified Eagle medium (DMEM; Thermo Scientific, South Logan, UT). Samples were washed 6 times in sterile PBS supplemented with 1% penicillin/ streptomycin/ amphotericin B (GIBCO, Grand Island, NY) and the subcutaneous fat was removed. Small pieces of minced skin were incubated in Dispase (25μg/ml in KSFM) (GIBCO) for 2hrs at 37°C to separate the epidermis from dermis. The dermal part was further minced into <3mm pieces and distributed into 60x15-mm Petri dishes. DMEM/10% FBS/1%antibiotic-antimycotic was added to the Petri dish and incubated at 37° C +5% CO_2. The culture medium was replaced every 3 days and proliferation of fibroblasts was monitored. Upon reaching 80-90 percent confluency, the fibroblasts were trypsinized and sub-cultured at a ratio of 1:5 in 75-cm^2 tissue-culture flasks.

1.2 Evaluation of the stability of encapsulated MTX and released from microspheres in vitro.

Fibroblasts were seeded in 25-cm^2 tissue culture flasks with a population of 5 x10^5 cells/flask and were treated with 50 ng/mL of freshly made MTX solution, MTX released from microspheres, and equal volume of PBS containing empty microspheres. After 48 hours the cells were harvested and lysed with lysis buffer (50 mM Tris-HCl, 150 mM NaCl, 1 mM EDTA, 1 mM EGTA, 0.025% NaN_3, 1% Triton-X 100, 0.5% IGEPAL CA-630, and 1:100 protease inhibitor cocktail from SIGMA). Cell debris was removed by centrifugation at 14,000 rpm at 4 °C for 10 min. After measurement of the protein concentration in the supernatant by Bradford protein assay, equal amounts of protein were loaded on 10% (w/v) acrylamide gel. After fractioned by SDS-PAGE, the proteins were transferred to a PVDF membrane (Millipore Corp., Bedford, MA). Non-specific bindings were blocked with 5% skimmed milk in Tris buffered saline containing 0.5%Tween-20 (TBST) overnight at 4°C and immunoblotting was performed using either anti-human procollagen type1α1 (1:100 dilution), or anti-MMP-1 (1:2000 dilution), or anti-β-actin (1:25,000 dilution). After five washes with TBST, the membranes were then incubated with the proper HRP conjugated secondary antibodies and immunoreactive proteins were then detected using enhanced chemiluminescence detection system (ECL; Santa Cruz Biotechnology, Santa Cruz, CA). The quantity of the proteins was determined by densitometry using ImageJ 64 software (Research Service Branch, national Institutes of Health).

1.3 Quantification of Collagen

Lyophilized whole PVA sponges, separated from overlying skin, were cut into small pieces, and incubated for 5–6 hours with one mL proteinase K (in Tris-HCl buffer) at 54 °C while shaking. Then, the mixture was

centrifuged and the PVA pieces were removed. The aliquots of the resultant homogenates were incubated with an equal volume of 12 N HCl at 105 °C overnight to hydrolyze the collagen. After evaporation of the acid under a nitrogen flow the dried samples were suspended in 40 μL of ethanol: d H_2O: TEA (2:2:1) and dried again. Samples were derivatized to the phenylthiocarbamyl form by adding 40 μL of ethanol: dH_2O: TEA: PITC (7:1:1:1) and incubating at room temperature for 20 min before drying. Resuspended samples in one mL of analysis solution (dH_2O: Acetonitrile, 7:2) and cleared were analyzed by HPLC. In addition, using the same procedure, one mg of purified type I bovine collagen was hydrolyzed and derivatized as control. Different amounts of hydroxyproline (1-320 μg) were dried from freshly prepared stock solutions of trans-4-hydroxy-L-proline in distilled water and derivatized same as the samples for the calibration curve.

1.4 ***Determination of α-SMA Protein Expression***

Formalin-fixed, paraffin-embedded tissue sections were deparaffinized and rehydrated. Heat-induced epitope retrieval was achieved with sodium citrate buffer (10 mM, pH 6.0) + 0.05% Tween 20. Then, sections were washed with tris-buffered saline (TBS) (20 mM, pH 7.4) and blocked with 5% normal goat serum + 5% bovine serum albumin in TBS. Following overnight incubation with rabbit polyclonal antibody to α-SMA at 4°C, sections were washed with TBS and incubated with 3% H_2O_2 at room temperature for 15 minutes. After one hour of incubation with the biotinylated goat anti-rabbit antibody as secondary antibody, sections were washed and incubated with a mixture of avidin-biotin from the Vectastain ABC kit, and the colored product of the enzyme horseradish peroxidase (HRP) was developed with the NovaRED substrate kit for peroxidase. The nuclei were counterstained with hematoxylin.

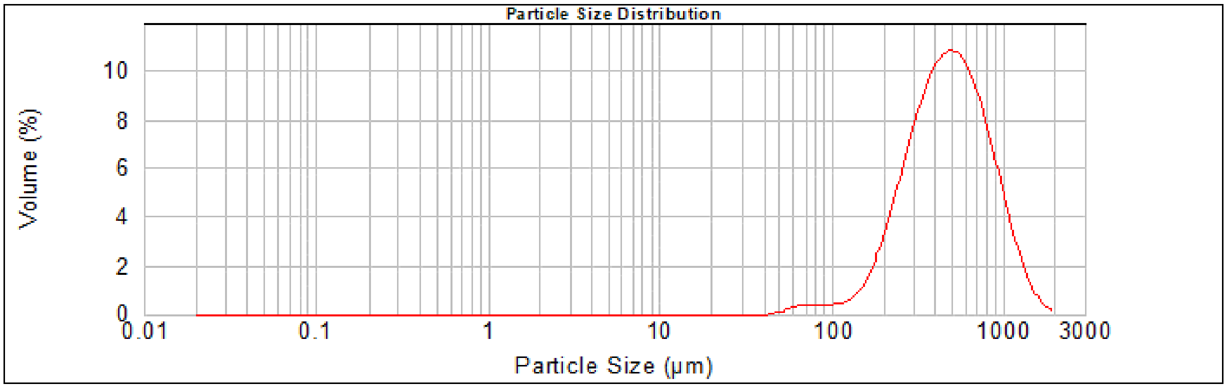

Supplementary Figure 1. Representative graph of particle size distribution on PLGA 75%+20%diblock Microspheres.
